# Optimization of the Properties of Photocured Hydrogels for Use in Electrochemical Capacitors

**DOI:** 10.3390/polym13203495

**Published:** 2021-10-12

**Authors:** Piotr Gajewski, Aneta Lewandowska, Katarzyna Szcześniak, Grzegorz Przesławski, Agnieszka Marcinkowska

**Affiliations:** Institute of Chemical Technology and Engineering, Poznan University of Technology, Berdychowo 4, 60-965 Poznan, Poland; aneta.b.lewandowska@doctorate.put.poznan.pl (A.L.); katarzyna.szczesniak@put.poznan.pl (K.S.); grzegorz.przeslawski@doctorate.put.poznan.pl (G.P.)

**Keywords:** hydrogel polymer electrolyte, photopolymerization, methacrylate, electrochemical capacitor, optimization

## Abstract

In this work, hydrogel polymer electrolytes (HPEs) were obtained by the photopolymerization of a mixture of two monomers: Exothane 8 (Ex8) and 2-hydroxyethylmethacrylate acid phosphate (HEMA-P) in an organic solvent N-methyl-2-pyrrolidone (NMP), which was replaced after polymerization with water, and then with the electrolyte. The ratio of monomers as well as the concentration of NMP was changed in the composition to study its influence on the properties of the HPE: conductivity (electrochemical impedance spectroscopy, EIS) and mechanical properties (puncture resistance). Properties were optimized using a mathematical model to obtain a hydrogel with both good mechanical and conductive properties. To the best of our knowledge, it is the first publication that demonstrates the application of optimization methods for the preparation of HPE. Then, the hydrogel with optimal properties was tested as a separator in a two-electrode symmetric AC/AC pouch-cell. The cells were investigated by cyclic voltammetry galvanostatic charge/discharge with potential limitation and EIS. Good mechanical properties of HPE allowed for obtaining samples of smaller thickness while maintaining very good dimensional stability. Thus, the electrochemical capacitor (EC) resistance was reduced and their electrochemical properties improved. Moreover, photopolymerization kinetics in the solvent and in bulk by photo-DSC (differential scanning calorimetry) were performed. The great impact on the polymerization of HEMA-P and its mixtures (with Ex8 and NMP) have strong intermolecular interactions between reagents molecules (i.e., hydrogen bonds).

## 1. Introduction

The development of civilization and the related increase in the demand for electricity forces us to look for new solutions in the field of energy storage devices [1,2,3]. Electrochemical capacitors (ECs), also known as supercapacitors, are one of the types of equipment that has been given special attention. Thanks to their specific properties, ECs can be applied everywhere where many fast charge/discharge cycles or peak power generation are required. To improve the performance of ECs, particular importance is attributed to each of their components–electrode materials, electrolytes, and separators [1]. One of the key parts of EC is the electrolyte. It can have a large impact on EC properties such as electrochemical stability window, capacity, thermal stability, energy, and power density, life cycle, etc. [2,3]. Taking into account the cost of production and ecological aspects, it seems that water based electrolytes are very promising for their application in ECs [1]. Additionally, these electrolytes display lower viscosity and higher conductivity in comparison to organic ones and ionic liquids as well as they are ecologically friendly and nonflammable [4,5,6].

Nowadays, much attention is given to the hydrogel polymer electrolytes (HPE), which simultaneously plays the role of an electrolyte, allowing for the fast transport of ions during charging/discharging and a separator to prevent direct contact between the electrodes and avoid short circuits [7,8]. Taking these requirements into consideration, the HPE should be characterized by high conductivity and high mechanical properties at the same time to provide the lowest possible ion transport resistance without losing dimension stability.

In the literature, various polymers have been used for the preparation of HPE such as polyvinylpyrrolidone (PVP), potassium polyacrylate (PAAK) [9], polyvinyl alcohol (PVA) [10,11,12], and others [13,14,15,16]. However, most of them have been mainly developed with a basic or acidic electrolyte or with KCl, NaCl, and LiCl neutral aqueous solutions that highly reduce the maximal cell potential to 0.8–1 V (these electrolytes are not stable above 1 V due to the evolution of hydrogen, oxygen, or chloride, respectively) [17,18,19]. The potential of ECs can be broadened up to 1.6 V by applying neutral electrolytes composed of sulfate or nitrate salts, but the number of papers on HPE with these salts is highly limited [12]. Additionally, most papers presented in the literature do not describe the mechanical properties of HPE or use HPE with high thicknesses, which considerably reduces the volumetric capacitance of ECs.

In addition, the preparation of HPE by the crosslinking of the ready polymer highly limits the possibility of HPE structure modification. Therefore, polymerization using monomers with various structures allows for the precise design of HPEs and their final properties. It seems that the best method of polymer matrix preparation is photopolymerization [15]. This process is very fast (a few minutes) and no by-product is obtained. Moreover, the use of UV radiation to initiate the polymerization process allows one to perform it at ambient temperature with low energy consumption. Therefore, photopolymerization seems to be the best choice.

To meet the requirements for hydrogel polymer electrolytes for their application in electrochemical double layer capacitors (EDLC), it is necessary to design their composition and properties in order to obtain high ionic conductivity (fast ion transport through HPE) and high mechanical properties at the same time. Therefore, proper design of a photocurable mixture composition is a crucial step of HPE synthesis because then the best compromise between mechanical properties and conductivity can be obtained. In the literature, the lack of information about the optimal selection of HPE composition are presented. Therefore, we decided to apply an optimization method to calculate a photocurable mixture composition to obtain the best connection between conductivity and mechanical properties of our HPE.

## 2. Materials and Methods

### 2.1. Materials

Monomers: Two-functional aliphatic urethane acrylate Exothane 8 (Ex8) and mono-functional 2-hydroxyethylmethacrylate acid phosphate (HEMA-P, HP) were delivered by ESSTECH (Esstech Inc., Essington, PA, USA) and 2-hydroxymethacrylate (HEMA) was delivered by Sigma-Aldrich (Sigma-Aldrich, St. Louis, MO, USA). Monomers were used without further processing. N-methyl-2-pyrrolidone (NMP), purity ≥ 99%; sodium sulfate (Na_2_SO_4_), purity ≥ 99%; sodium hydroxide (NaOH), purity ≥ 98% and photoinitiator, 2,2-dimethoxy-2-phenylacetophenone (DMPA) were provided by Sigma-Aldrich (Sigma-Aldrich, St. Louis, MO, USA).

### 2.2. Methods

#### 2.2.1. Photopolymerization Kinetics

The photopolymerization kinetic was monitored by isothermal differential scanning calorimetry (photo-DSC) using a Pyris 6 instrument (Perkin–Elmer, Waltham, MA, USA) equipped with a lid especially designed for photochemical measurements. The 2 ± 0.2 mg samples were polymerized in open aluminum pans (diameter 6.6 mm) under isothermal conditions (25 °C) in a high-purity argon atmosphere (<0.0005% of O_2_). Before and during the polymerization reaction, the chamber of the apparatus was purged with an inert gas flowing at a rate 100 mL·min^−1^. The polymerization was initiated by the UV light (365 nm, light intensity 1 mW·cm^−2^) from the LED lamp (LC-L1, Hamamatsu Photonics, Hamamatsu, Japan). All photopolymerization experiments were conducted at least in triplicate. The reproducibility of the kinetic results was about ± 3%. For calculation, the heat of the polymerization of the methacrylate group, 56 kJ·mol^−1^, was taken [20]. The compositions used for kinetic measurements are listed in Table 1. Additionally, compositions containing HP or Ex8 with 50, 60, 70, 80 wt.% of NMP and mixtures of monomers Ex8: HP (in weight ratios 30:70, 40: 60, 50:50, and 60:40) without the addition of solvent were prepared.

#### 2.2.2. Characterization of Reagents

Viscosity: Viscosities of reagents were measured by a DV-II+ PRO Brookfield Rheometer in the cone-plate configuration at 25 °C.

FTIR spectroscopy: Infrared spectra were performed on a Nexus Nicolet 5700 Fourier transform infrared spectrophotometer (FTIR, Thermo Electron Scientific Instruments Corporation, Madison, WI, USA) at room temperature in a wavenumber range 4000–600 cm^−1^ and resolution of 4 cm^−1^ at 64 scans. The tested composition was placed as a thin film between two NaCl plates.

#### 2.2.3. Hydrogel Preparation

**Figure 1:**
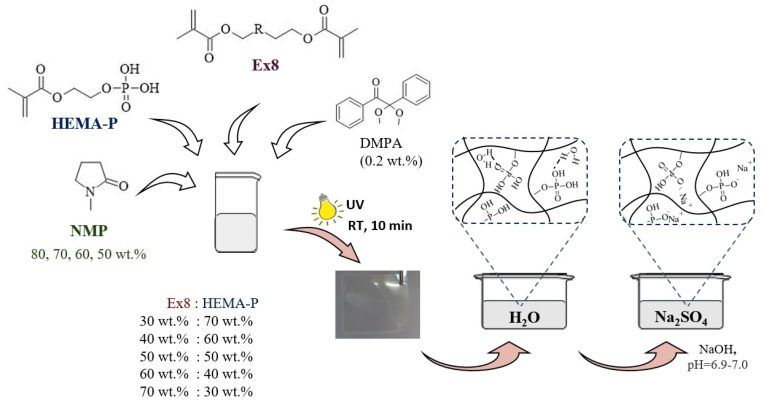
Scheme of hydrogel polymer electrolyte preparation.

The photocurable compositions consisted of a mixture of monomers, solvent NMP, and photoinitiator DMPA (0.2 wt.% on the whole composition) in appropriate mass fraction (Table 1) were mixed in an orbital shaker until homogeneous solutions were obtained (Figure 1). Then, the selected composition was poured into a 0.3 mm thick glass mold and exposed at room temperature to UV light for 10 min on each side of the mold (ASN-36W UV lamp, light intensity 6 mW·cm^−2^). After polymerization, all samples were placed in distillated water for 24 h to replace NMP with water. Then, samples were placed in 1 M Na_2_SO_4_ solution to replace water with the electrolyte. During that step, a solution of NaOH was added to maintain the pH = 6.9–7.0. After the electrolyte sorption equilibrium was reached, the hydrogel polymer electrolytes (HPEs) were obtained and test samples with certain dimensions were cut from them. As a criterion of equilibrium, a constant mass of HPE was taken. In the next two measurement of the sample mass, with an interval of three hours, the difference in the mass must be less than 2%, at a constant pH = 6.9–7.0. A scheme of the preparation of hydrogels is shown in Figure 1.

#### 2.2.4. Water and Electrolyte Sorption

Study of water and electrolyte sorption for five samples of hydrogel (after water sorption) and hydrogel polymer electrolyte (after sorption of 1 M Na_2_SO_4_) were performed. Before the samples were weighed, excess water from the surface was removed with tissue paper. Then, samples were dried for a 3 h in 100 °C and again were weighed. Based on obtained results, the mass fraction of water (X_wt,H2O_) and electrolyte (X_wt,el_) were calculated from Equation (1):

X_wt,H2O_ or X_wt,el_ = (m_gel_ − m_dry_)/m_gel_, (1)

where X_wt,H2O_ and X_wt,el_ is the mass fraction of water and electrolyte, respectively; m_gel_ is the weight of hydrogel or hydrogel polymer electrolyte, respectively; and m_dry_ is the mass of the dry polymer (after 3 h of drying).

#### 2.2.5. Scanning Electron Microscope (SEM)

The morphology of the studied samples were investigated using a scanning electron microscope JEOL 7001F (JEOL Ltd., Tokyo, Japan) (SEI detector, 15 kV accelerating voltage). Small pieces of each sample of hydrogel after preparation (i.e. photopolymerization, water, and electrolyte sorption, and drying in laboratory oven at 100 °C) were placed on a stub of metal with adhesive and then coated with an ultrathin gold coating, deposited on the samples by low-vacuum sputter coating.

#### 2.2.6. Puncture Resistance

In order to characterize the mechanical properties of the obtained HPE, a puncture resistance test was conducted similarly to the procedure described in our previous paper [21]. Briefly, the measurements were performed with a CT3 Texture Analyzer (Ametek Brookfield). The mechanical properties of the obtained HPE were measured after electrolyte sorption. The sample was fixed in a sample holder and tested for puncture strength using the spherical probe with 5 mm diameter. During the measurement, the force and distance of the measuring probe were recorded until the sample was punctured (probe displacement rate–0.3 mm·s^−1^). Because different HPE slightly differ in their thickness, the force was normalized to a uniform thickness (250 µm) in order to compensate for the influence of thickness on the measurement. For each HPE, the experiment was repeated for five or six samples. Based on the obtained results, the means and standard deviations were calculated. 

#### 2.2.7. Ionic Conductivity

The ionic conductivity of the HPE was investigated by electrochemical impedance spectroscopy (EIS) in the frequency range from 1 kHz to 1 MHz using the SP-300 potentiostat/galvanostat (Biologic) in accordance with the procedure described previously [21]. The experiment was performed in a two-electrode (stainless steel-316L) type electrochemical vessel at room temperature. The ionic conductivity of the HPE (σ) was calculated from Equation (2):

σ = l/(A × σ_s_) (2)

where σ is the ionic conductivity of the HPE-S·cm^−1^; l is the thickness of the HPE-cm; A is a HPE surface area—cm^2^, and σ_S_ represents the volumetric conductance of the HPE sample-S.

For each HPE, six measurements of conductivity were made. Based on the obtained results, the means and standard deviations were calculated. 

#### 2.2.8. Optimization of Hydrogel Polymer Electrolyte Composition

Application of hydrogels as polymer electrolytes in ECs require both good conductive and mechanical properties. For that purpose, a mathematical model with the highest order (cubic model, Equation (3)) was fitted to the data of puncture resistance and conductivity and then deleted one term each, starting with the highest *p*-value (backward elimination procedure). This was continued until all remaining terms had a significant *p*-value (*p* < 0.05).

y = *b*_1_·X_NMP_ + *b*_2_·X_HEMA-P_ + *b*_3_·X_Ex8_ + *b*_12_·X_NMP_·X_HEMA-P_ + *b*_13_·X_NMP_·X_Ex8_ + *b*_23_·X_HEMA-P_·X_Ex8_ + *d*_12_·X_NMP_·X_HEMA-P_·(X_NMP_ − X_HEMA-P_) + *d*_13_·X_NMP_·X_Ex8_·(X_NMP_ − X_Ex8_) + *d*_23_·X_HEMA-P_·X_Ex8_·(X_HEMA-P_ − X_Ex8_) + *b*_123_·X_NMP_·X_HEMA-P_·X_Ex8_ (3)

where *b*_1_, *b*_2_, *b*_3_, *b*_12_, *b*_13_, *b*_123_, *b*_23_, *d*_12_, *d*_13_, and *d*_23_ are the model parameters and X_NMP_, X_HEMA-P_, and X_Ex8_ are mass fraction of components in photocurable composition.

After determining the appropriate model describing the dependence of the puncture resistance (*y_p_*) and conductivity (*y_c_*) of HPE on the photocurable composition, the objective function was defined as a product of *y_p_* and *y_c_,* and photocurable composition at maximum of objective function was determined. All calculations were undertaken in Statistica software (TIBCO Software Inc., Palo Alto, CA, USA).

#### 2.2.9. Electrochemical Measurements of Capacitors

##### Preparation of Electrodes

The carbon electrodes were prepared by mixing the appropriate amount of activated carbon—90 wt.% of Maxsorb MSP-20X (Kansai Coke and Chemi-calsco) with carbon black—5 wt.% of C65 (Imerys) and binder—5 wt.% of PTFE (60 wt.% water suspension, Sigma-Aldrich). The electrode components were mixed in deionized water until a homogenous suspension was obtained. Then, the solvent was partially evaporated and a thin film was prepared by rolling out and calendaring to an average thickness of 200 ± 15 μm. Thereafter, the electrode film was stuck on an etched stainless steel current collector coated with Acheson electrodag PF-407C (Henkel).

##### Electrochemical Investigations

The influence of the HPE on the properties of the electrochemical capacitor was evaluated in a two-electrode symmetric AC/AC pouch-cell. Before assembling the pouch-cell, the 20 mm × 25 mm carbon electrodes were soaked off in a 1M Na_2_SO_4_ and the 25 mm × 30 mm HPE was used as a separator and electrolyte. The mass of all electrodes used in the ECs construction was in the range of ca. 72–75 mg, therefore, the mass of activated carbon in the electrodes was about ca. 65-68 mg. Later average AC mass in one electrode was used for current density and specific capacitance calculation. The cells were investigated by cyclic voltammetry (CV) with various scan rates from 5 to 100 mV·s^−1^ and up to maximal cell potentials from 1 to 1.5 V, galvanostatic (0.5 to 5 A·g^−1^, calculated per average AC mass in one electrode) charge/discharge with potential limitation (GCPL), long cycling performance (2000 cycles of charging/discharging from 0 to 1.5 V at 0.5 A·g^−1^), and electrochemical impedance spectroscopy at open circuit voltage (OCV) (EIS, over the frequency range from 1 MHz to 1 mHz with a 10 mV amplitude) using an SP-300 potentiostat/galvanostat (Biologic, France).

## 3. Results

### 3.1. Characterization of Reagents

**Figure 2:**
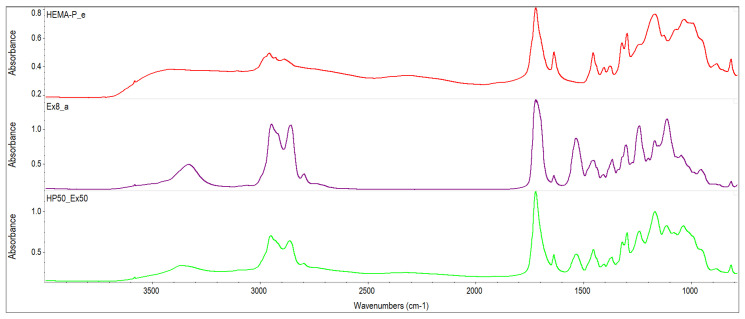
FTIR spectra of investigated monomers HEMA-P and Ex8 and equilibrium mixture of monomers.

**Figure 3:**
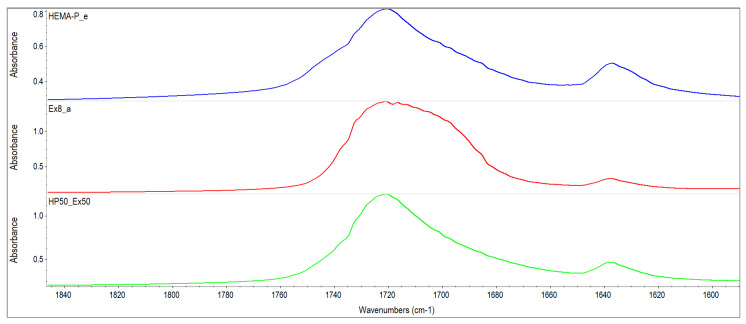
FTIR spectra of investigated monomers HEMA-P and Ex8 and equilibrium mixture of monomers in wavenumber range of 1850–1590 cm^−1^.

The monomer mixtures prepared in the NMP solvent were miscible in the entire concentration range. On the other hand, monomer mixtures without the addition of a solvent were mixed in a limited concentration range (i.e. compositions containing up to 60% of Ex8 in HEMA-P gave homogeneous mixtures). Therefore, FTIR spectra of monomers and their mixture in equilibrium ratio 50Ex:50HP were taken and shown in Figure 2. The absorption of HEMA-P in the P–O–H stretching region about 3400–3300 cm^−1^ consists of several overlapping bands, which can be ascribed to OH–C=O and OH–OH representing strong hydrogen bonds between its molecules. In this region, free hydroxyl groups and overtones of C=O also occur. Interactions of OH–C=O type are also reflected in the C=O stretching region at 1700 cm^−1^ consisting of free C=O groups and C=O involved in C=O–H–O bonding. The absorption of Ex8 in the C=O stretching region about 1700 cm^−1^ is composed of overlapping bands that can be attributed to free carbonyl groups and N–H–O=C type of hydrogen bond. Whereas the band of absorption of the N–H group occurs in the region 3300 cm^−1^. Intermolecular interactions in the reagents occur mainly between P–OH or C=O groups from HEMA-P and N–H or C=O groups from Ex8. In Figure 3, the spectra in the range of C=O group absorption bands for monomers and 50Ex:50HP system are shown. Addition of HEMA-P to Ex8 causes some changes in the shape of the C=O absorption band. The changes in the C=O stretching region confirm the disappearance of NH–C=O interactions in Ex8, and O–H–O=C in HEMA-P molecules. A similar effect was observed in the case of the spectrum region 3400–3000 cm^−1^, which can be related to the disappearance of hydrogen bonds in the NH and OH groups of monomers. 

Additionally, the viscosity of the studied compounds was examined: monomers and solvent, which are shown in Table 2.

### 3.2. Photopolymerization Kinetic

**Figure 4:**
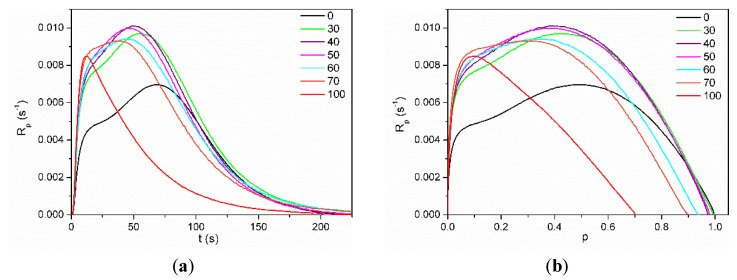
Dependence of polymerization rate (Rp) on (**a**) polymerization time (t) and (**b**) double bond conversion (p) for mixtures of HEMA-P with Ex8 in 50 wt.% of NMP concentration. Numbers on graphs indicate NMP concentration in the investigated systems.

Kinetic curves as a dependence of polymerization rate (R_p_) on the degree of conversion (p) for systems containing 50 wt.% of solvent N-methylpyrrolidone (NMP) are shown in Figure 4. 

**Figure 5:**
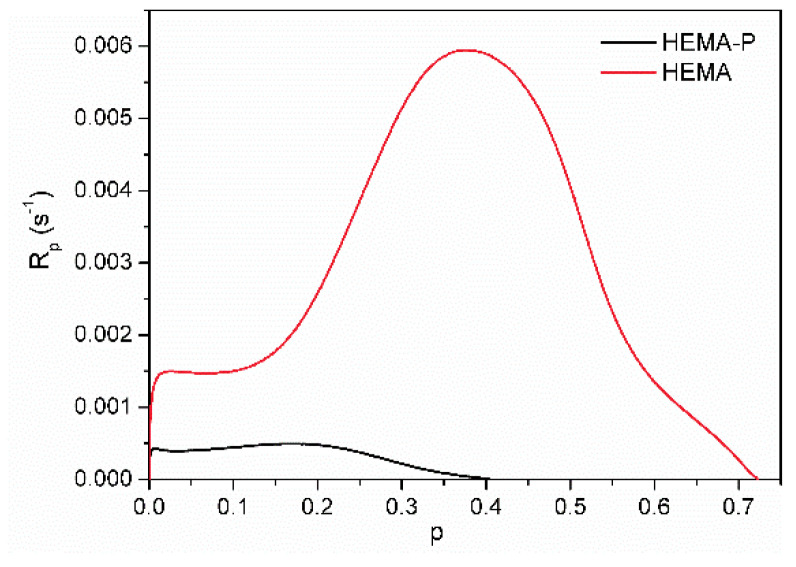
Kinetic curves of the photopolymerization of HEMA and HEMA-P.

The solvent polymerization (in NMP) of dimethacrylate monomer Exothane 8 (tetrafunctional monomer) was carried out with a higher reaction rate than monomethacrylate monomer HEMA-P (difunctional monomer) and has a characteristic shape for the polymerization of multifunctional monomers with immediate autoacceleration. On the other hand, the polymerization of HEMA-P proceeds with the gel effect (after approx. 20% of double bond conversion), characteristic of the linear polymerization of difunctional monomers. Monomer mixtures HEMA-P with Ex8 polymerize with a higher maximum polymerization rate (R_p_^max^) than neat monomers. Moreover, a composition (containing 40 wt.% of Ex8) with the highest value of R_p_^max^ appeared. As the concentration of the Ex8 in the investigated systems increased, the gel effect gradually disappeared, but at the same time, the initial rate of polymerization (before the steady-state) increased. This is related to the increase in the concentration of the cross-linking monomer in the investigated systems, therefore with a higher crosslinking density of the resulting polymer. The formation of a polymer network causes autoacceleration to occur earlier, hence the disappearance of the gel effect and an increase in the initial rate of polymerization. The final conversion of double bonds p_f_ decreases with increasing Ex8 concentration in the monomer mixture. As can be seen from Figure 4b, HEMA-P in 50 wt.% of NMP polymerizes with 100% conversion, but Ex8 only with 70%. For compositions containing 0–50 wt.% of Ex8, only a slight decrease of p^f^ was observed, but for compositions with higher Ex8 content (60 and 70 wt.%), this decrease was more pronounced. This result is probably related to the strong intermolecular interactions (i.e. hydrogen bonds) present in the HEMA-P monomer, as mentioned earlier in Section 3.1. *Characterization of Reagents*. To confirm this assumption, polymerization kinetic for systems without the addition of the NMP solvent was investigated. Neat HEMA-P monomer polymerizes with a very low rate and with a low final double bond conversion, approx. 41% (Figure 5). In contrast, polymerization of a classical HEMA monomer (2-hydroxymethacrylate) goes with a much higher polymerization rate and up to 1.8 times higher conversion due to the strong gel effect during polymerization. 

**Figure 6:**
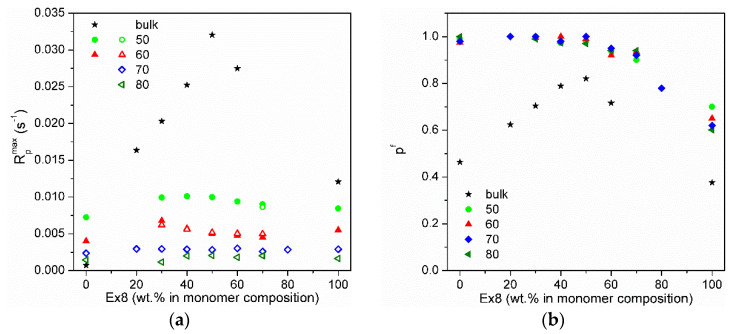
(**a**) Maximum polymerization rate (R_p_^max^): full points—maximum polymerization rate in gel effect, and empty points—in initial polymerization rate and (**b**) final double bond conversion (p^f^) on Ex8 content in the monomer mixture. Numbers indicate NMP concentration in the composition in wt.%.

HEMA-P, due to the presence of the phosphate group in its structure, forms strong intermolecular hydrogen bonds, resulting in a quasi-network hydrogen bonded structure. This structure results in high viscosity (578 mPa·s) of the monomer (two orders of magnitude larger than HEMA viscosity) and hindered reaction environment, which must be responsible for the small conversion and polymerization rate achieved by this monomer. Hydrogen bonding of the monomer molecules can cause the monomer to behave as a multifunctional monomer and results in the formation of a highly cross-linked structure that limits polymerization. On the other hand, the addition of Ex8 causes the destruction of these quasi-network hydrogen bonded structures, and therefore, HEMA-P can diffuse more freely in the system, which leads to an increase in the polymerization rate (Figure 6a) and final degree of double bond conversion (Figure 6b). On the other hand, the addition of HEMA-P to Ex8 caused a decrease in the initial monomer viscosity and a decrease in the cross-linking density, which reduced the diffusion limitations during polymerization.

The dependence of maximum polymerization rate (R_p_^max^) and final conversion of double bonds (p^f^) on Ex8 concentration in investigated systems for different solvent content are shown in Figure 6a,b, respectively. The addition of NMP solvent to HEMA-P caused an increase in the reaction rate as well as the final conversion of double bonds because of the destruction of hydrogen bonds between the monomer molecules and decrease in mixture viscosity. Polymerization of Ex8 showed the classical influence of the solvent on polymerization (dilution effect) (i.e. a decrease in the polymerization rate). In addition, an increase in the final p^f^ conversion due to a decrease in the viscosity of the starting composition and growth during polymerization, and thus the delay in the occurrence of diffusion limitations of polymerization was observed. A classical solvent effect of NMP also occurs in the copolymerization of the monomer mixtures (i.e. gradual disappearance of the gel effect (decrease in the maximum polymerization rate) with increasing dilution of monomer mixture). The final double bond conversion increases for systems with NMP, indicating the plasticizing effect of the solvent.

### 3.3. Electrolyte Sorption and Conductivity

**Figure 7:**
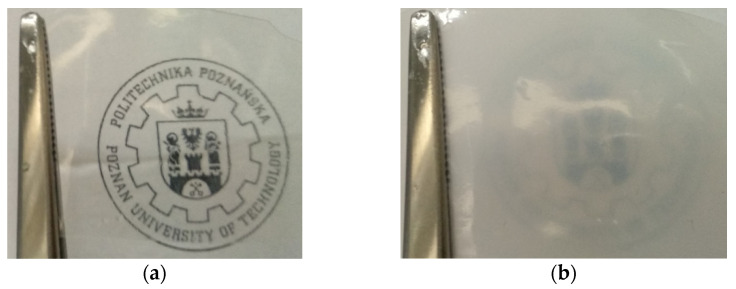
Pictures of materials obtained by the photopolymerization of the composition containing equilibrium mixture of monomers with 50 wt.% of NMP (5Ex:5HP_50) (**a**) after polymerization, (**b**) after electrolyte sorption.

Photopolymerization of the investigated compositions of monomer mixtures with different solvent content (50–80 wt.%) were performed. Obtained materials were homogenous and completely transparent. After electrolyte sorption, hydrogels obtained with 50 wt.% of NMP, regardless of the weight ratio of the monomers, partially became opaque (Figure 7). 

**Figure 8:**
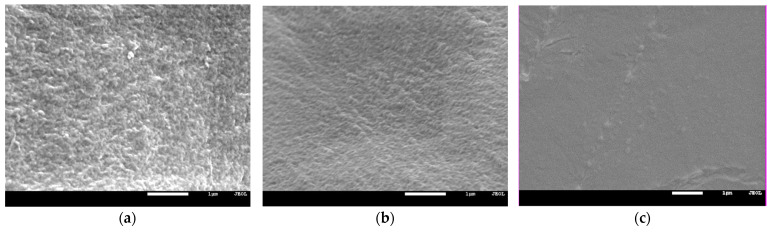
SEM images of the polymer matrix 7Ex:3HP with (**a**) 50, (**b**) 60, and (**c**) 70 wt.% of NMP.

The influence of the amount of solvent in the initial composition on the structure of the polymer matrix was confirmed by the SEM images (Figure 8). As can be seen from Figure 8, an increase in the solvent content contributes to an increase in the homogeneity of the matrix. The monomers used in the photocurable composition show limited miscibility, as mentioned in Section 3.1. Characterization of Reagents. Thus, the obtained results indicate intermolecular interaction improvement between components of the composition with increasing NMP concentration. 

**Figure 9:**
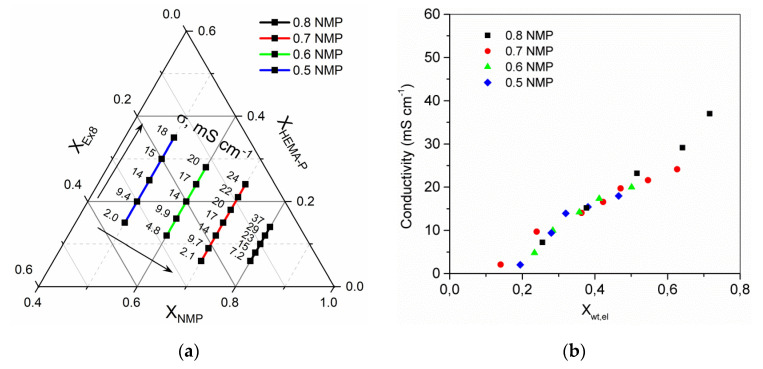
(**a**) Ternary plot of HPE ionic conductivity dependence on photocurable mixture composition, the values presented in ternary plot are the ionic conductivity in mS·cm^−1^. (**b**) Dependence of HPE conductivity on mass fraction of electrolyte in HPE. Conductivity of 1 M Na_2_SO_4_ electrolyte σ_el_ = 80 mS·cm^−1^.

Next, the properties of the obtained HPE were examined. The dependence of conductivity (Figure 9a) and electrolyte mass fraction in HPE (Figure S1a ESI) on a photocurable mixture composition is presented. It could be seen that both the solvent and monomer mass fraction in the mixture have an important influence on obtained results. The electrolyte mass fraction and conductivity of HPE increase with increasing mass fraction of the solvent (compositions where mass ratio of monomers is constant) and increase with increasing mass ratio of monomer HEMA-P to Ex8 (for constant mass fraction of solvent). The arrows, on the plot, indicate the direction of progressive increase in the values. Obtained results of conductivity and electrolyte sorption confirm that these two parameters are correlated (Figure 9b). As could be expected, higher conductivity was observed for HPE with higher mass fraction of electrolyte after sorption. 

By changing a photocurable mixture composition, it is possible to regulate the conductivity of HPE in a broad range from ca. 2 mS·cm^−1^ up to ca. 40 mS·cm^−1^. This is possible thanks to the modification of the solvent concentration in the photocurable mixture and application of monomers with different properties. HEMA-P, in contrast to Ex8, is a highly polar monomer, which can strongly interact and easily absorb electrolyte solution. Therefore, increasing the content of HEMA-P at the cost of Ex8 causes a fast increase in electrolyte sorption and increase in conductivity. The influence of NMP on electrolyte sorption and conductivity is more complex. Increasing mass fraction of solvent considerably influences the spatial structure of the obtained polymer. The greater the solvent, the greater the free space between the chains of the obtained polymer. Therefore, a decrease in cross-linking density of the polymer network can be expected, which increases the absorption of the electrolyte into the polymer structure and allows for HPE to be obtained with higher conductivity. A similar dependence could be observed between the mass fraction of water and the composition of the photocurable mixture (Figure S1b ESI), but for the same mixture compositions, a lower water absorption could be observed. This may be related to the physical cross-linking between the water and the HEMA-P functional groups due to the formation of hydrogen bonds. During electrolyte sorption, NaOH was added (to maintain the pH = 6.9–7.0), which neutralizes HEMA-P and the hydrogen bonds disappeared. As a result, the structure was no longer as densely cross-linked.

### 3.4. Mechanical Properties

**Figure 10:**
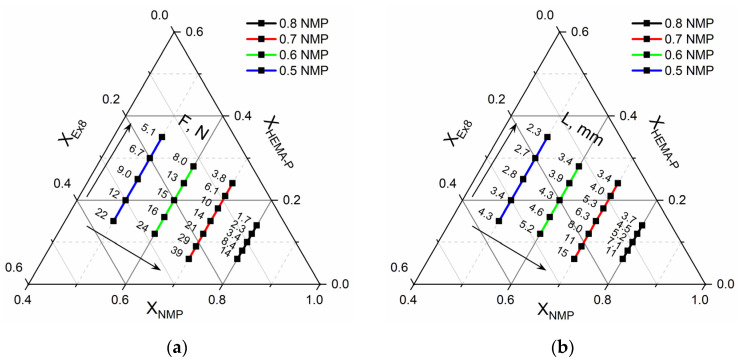
Ternary plot of (**a**) puncture resistance-F, N, and (**b**) elongation at puncture-L, mm dependence on mixture composition.

In Figure 10, the mechanical properties of the obtained HPE are presented. As can be seen, force necessary to puncture (Figure 10a) and elongation at puncture (Figure 10b) of the obtained HPE significantly depends on the composition of the photocurable mixture. The puncture resistance changed from ca. 2 N up to ca. 40 N. At the same time, the elongation at puncture changed from ca. 2 mm up to ca. 15 mm. It could be observed that increasing the ratio of Ex8 to HEMA-P caused a significant increase in both parameters. Such a behavior is connected with the properties of Ex8 resin, which is highly elastic and tough material after polymerization (elongation at break–79%, toughness–5.7 J, data from producent webpage) [22]. The influence of solvent on mechanical properties is more complex. It can be seen that increasing the mass fraction of solvent in a photocurable composition from 0.5 to 0.7 (keeping the same mass ratio of monomers) increases both the puncture force and elongation at puncture. This improvement in mechanical properties is less significant compared to the effect of the mass ratio of the monomers, but still clearly visible. For the largest mass fraction of the solvent, equal to 0.8, a decrease in both mechanical parameters could be observed. The decrease in mechanical properties of the samples with decreasing amounts of solvent was different from the expected behavior. This can be explained by the change in the polymer structure with the amount of solvent used, as can be seen from the SEM micrographs presented in Figure 8. The smaller the concentration of the solvent in the range 50–70 wt.%, the more heterogeneous the structure of the polymer matrix. This effect was also seen as a loss of transparency in the pictures presented in Figure 7. Hence, HPE with a 30 wt.% of polymer matrix showed better mechanical properties.

### 3.5. Optimization of the Photocurable Mixture Composition

Application of hydrogels as a polymer electrolyte in supercapacitors requires receiving HPE with high mechanical properties and high conductivity at the same. Only in that case, it is possible to design ECs characterized by high capacitance, even at high current. As presented in Figure 10a,b, by changing the photocurable mixture composition, it is possible to manage the mechanical properties and conductivity. However, changing the mixture composition to increase mechanical properties caused decreasing conductivity and reverse, increasing conductivity results in a decrease in mechanical properties. Therefore, it is important to find a compromise between these two parameters. For that purpose, the mathematical model was fitted to the data of puncture resistance and conductivity and the maximum of objective function was determined.

**Figure 11:**
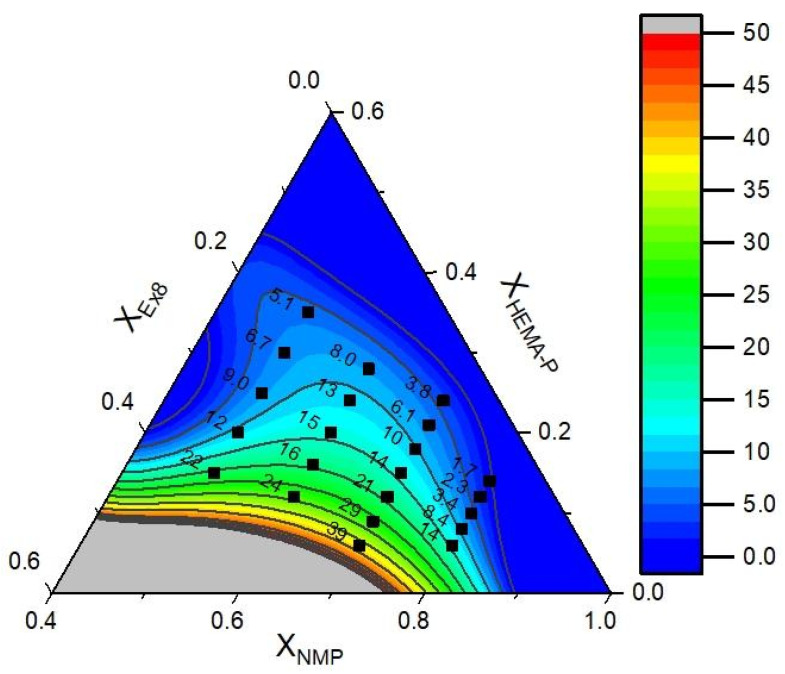
Experimental data of puncture resistance (dots) and fitted function (contours).

**Figure 12:**
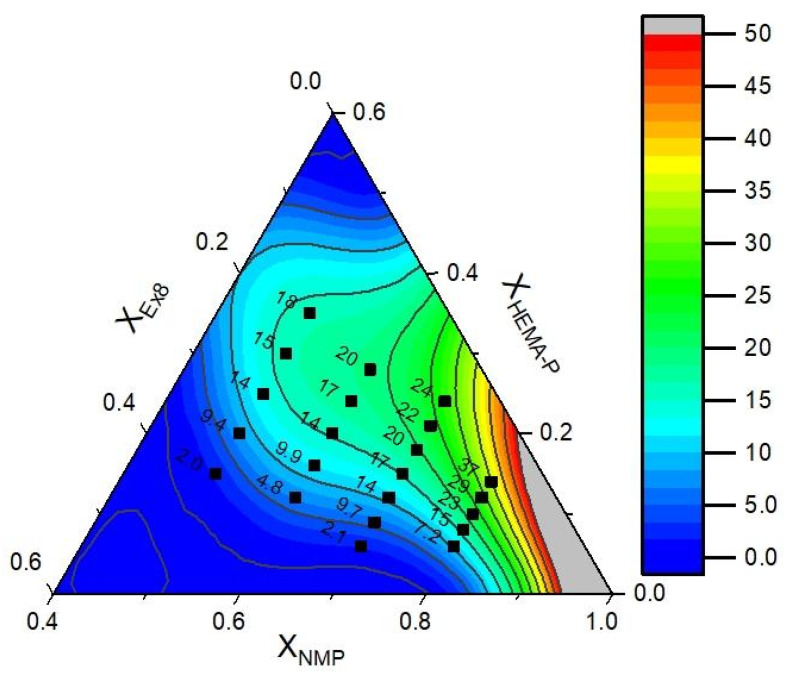
Experimental data of the conductivity (dots) and fitted function (contours).

The data of puncture resistance and conductivity with fitted mathematical function (contours) are presented in Figure 11 and Figure 12, respectively. In Table 3, the model parameters are presented. As could be seen, a very good approximation of experimental data by mathematical model was obtained. It can also be confirmed by the plot of predicted vs. actual value (Figure S2a,b, ESI). Therefore, in the next step, the objective function was defined as a product of *yF* and *yP* and the maximum of this function was determined (in order to obtain the maximum in the investigation area, the restriction to NMP mass fraction X_NMP_ = 0.5–0.8 was introduced). The above calculations allow us to obtain a photocurable mixture composition, which should be characterized by the best parameters (Table 4).

As can be seen (Table 4), puncture resistance and conductivity calculated from a mathematical model corresponded very well with the results obtained for the sample.

### 3.6. Electrochemical Investigation

Optimization of the photocurable mixture composition allowed us to obtain HPE with the best compromise between mechanical properties and conductivity (Table 4). Great mechanical properties allow us to prepare HPE with lower thicknesses, keeping very good dimensional stability. As a result, it is possible to reduce the resistance of HPE and thus improve the electrochemical properties of the EC. Therefore, we decided to synthesize HPE with two different thicknesses (95 ± 6 μm and 298 ± 18 μm, average thickness of HPE measured after electrolyte sorption, later marked as HPE100 and HPE300) to present the influence of the HPE resistance on the properties of ECs.

**Figure 13:**
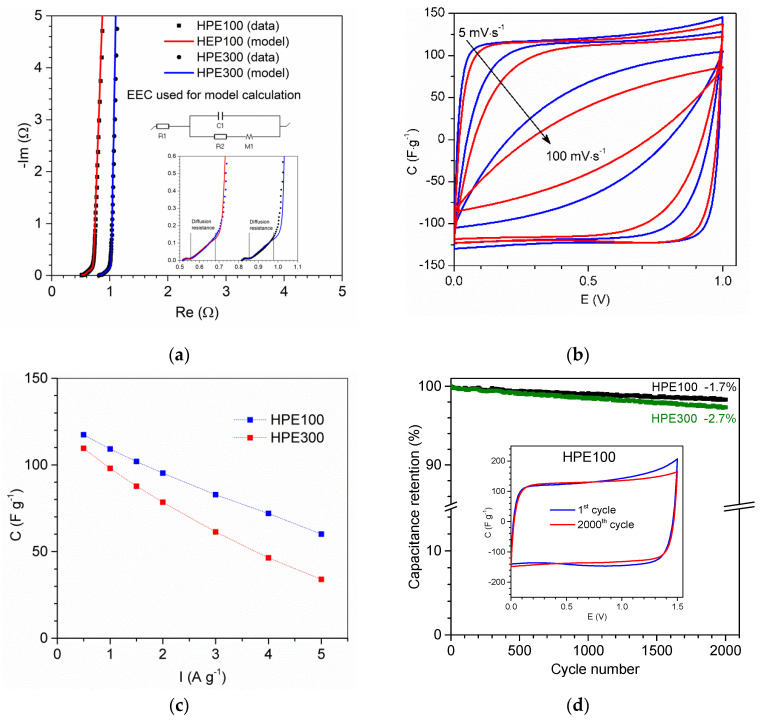
(**a**) Nyquist plot; (**b**) cyclic voltammograms at scan rates 5 and 100 mV·s^−1^, current value is converted to capacitance; (**c**) discharge capacitance vs current of AC/AC capacitors with applied HPE; (**d**) change of the relative capacitance calculated from constant current technique (0.5 A·g^−1^) and (inset) cyclic voltammograms after cyclability test, performed at 5 mV·s^−1^. Capacitance and current, expressed per AC mass in one electrode.

The Nyquist plot of the AC/AC capacitor (Figure 13a) shows that equivalent series resistance (ESR) of EC with applied HPEs was equal ca. 0.52 Ω (after area normalization 2.6 Ω·cm^−2^) and 0.82 Ω (after area normalization 4.1 Ω·cm^−2^) for HPE100 and HPE300, respectively. It can be seen that the difference in ESR value equal 1.5 Ω·cm^−2^ corresponds very well to the difference in the resistance of the HPE100 and HPE300 equal to 1.4 Ω·cm^−2^. This result clearly shows that by reducing the thickness of the HPE, the EC resistance can also be significantly reduced. Obtained data of impedance spectroscopy were modeled by equivalent electric circuit (EEC). As can be seen, the calculated model very well corresponded to the obtained results. Model parameters are presented in Table S1 ESI. Based on the results of modeling, it can be seen that capacitance C1, resistance R2, and parameters describing diffusion resistance are very similar for both capacitors. Only resistance R1, which corresponds to ESR, was different, which corresponds to different resistance of applied HPE.

The CV of the AC/AC capacitor (Figure 13b) obtained at voltages up to 1.0 V was close to the box-like shape of an electric double-layer capacitor (EDLC). The absence of peaks on the CV curve exhibited a capacitive behavior of EDLC without redox reaction. As can be seen, CV at a scan rate of 5 mV·s^−1^ for both ECs presented very similar shape of the curve and capacitance equal to 119 F·g^−1^ and 115 F·g^−1^ for HPE100 and HPE300, respectively. After increasing the scan rate up to 100 mV·s^−1^, we can clearly see that CV of EC with HPE100 was better. Capacitance of EC at a scan rate 100 mV·s^−1^ with HPE100 and HPE300 was equal 66 F·g^−1^ and 49 F·g^−1^, respectively.

In Figure 13c, the discharge capacitance vs. current is presented. As can be seen, current changing from 0.5 A·g^−1^ to 5 A·g^−1^ caused a decrease in capacitance from ca. 117 F·g^−1^ to ca. 60 F·g^−1^ and from ca. 110 F·g^−1^ to ca. 34 F·g^−1^ for EC with HPE100 and HPE300, respectively. Comparing the results obtained for capacitors with both HPE, we can clearly see that ECs presented similar capacitance/energy at a low value of current density or scan rate. Thus, with an increase in these parameters, we observed a greater difference in the obtained values of capacitance, with better results obtained for EC with HPE about lower thickness. Furthermore, we should consider that the lower thickness of HPE resulted in a lower thickness of EC and the same reduced the size of the device. All these results show that application of thinner HPE can significantly improve the electrochemical properties of ECs.

In Figure 13d, the results of the cyclic charge/discharge tests are presented. As can be seen, after 2000 cycles at 1.5 V, the capacitance of EC with HPE100 and HPE300 slightly decreased about 1.7% and 2.7%, respectively. Results obtained for EC with HPE100 presented very good cycle stability, which confirmed that EC with HPE100 used might operate at 1.5 V, and the discharge profile on the CV curve (Figure 13d inset) was almost the same after the 1st and 2000th cycles.

## 4. Conclusions

Application of the hydrogel polymer electrolyte (HPE) in electrochemical capacitors requires preparing HPE characterized by good mechanical properties as well as good conductivity, at the same time. We demonstrated that proper choice of photocurable mixture composition allowed us to regulate both of these properties of HPE. We were able to obtain HPE with conductivity, in the range from ca. 2.0 to ca. 40 mS·cm^−1^, puncture resistance from ca. 2 N up to ca. 40 N, and elongation at puncture from ca. 2 mm up to ca. 15 mm. On the basis of the obtained results and thanks to the use of the optimization method, we were able to determine the composition of the photopolymerization mixture, which allows for the preparation of HPE with the best properties. To the best of our knowledge, it is the first publication that demonstrates the application of optimization methods for the preparation of HPE. Thus, the obtained HPE was successfully applied in electrochemical capacitors in a neutral aqueous electrolyte. Good mechanical properties allow for a significant reduction in the HPE thickness while maintaining high dimensional stability. The lower thickness of HPE and thus the lower equivalent series resistance of EC leads to obtaining EC with better electrochemical properties. Both the addition of the cross-linking monomer Ex8 and the NMP solvent to difunctional monomer HEMA-P destroy the hydrogen bonds between the monomer molecules, leading to the gradual disappearance of the gel effect and an increase in the polymerization rate. Strong interactions between monomers cause limited miscibility between them, which is improved by the addition of NMP. This influences the structure of the obtained materials, the heterogeneity of which decreases with increasing solvent content in the photo-curable composition.

## Figures and Tables

**Table 1:** Formulations of the investigated compositions (Ex8 + HEMA-P)/NMP (with 0.2 wt.% of DMPA photoinitiator).

Formulation	Ex8	HEMA-P	NMP
	wt.%		
30Ex:70HP_50NMP	15	35	50
40Ex:60HP_50NMP	20	30	50
50Ex:50HP_50NMP	25	25	50
60Ex:40HP_50NMP	30	20	50
70Ex:30HP_50NMP	35	15	50
30Ex:70HP_60NMP	12	28	60
40Ex:60HP_60NMP	16	24	60
50Ex:50HP_60NMP	20	20	60
60Ex:40HP_60NMP	24	16	60
70Ex:30HP_60NMP	28	12	60
20Ex:80Ex_70NMP	6	24	70
30Ex:70HP_70NMP	9	21	70
40Ex:60HP_70NMP	12	18	70
50Ex:50HP_70NMP	15	15	70
60Ex:40HP_70NPM	18	12	70
70Ex:30HP_70NMP	21	9	70
80Ex:20HP_70NMP	24	6	70
30Ex:70HP_80NMP	6	14	80
40Ex:60HP_80NMP	8	12	80
50Ex:50HP_80NMP	10	10	80
60Ex:40HP_80NMP	12	8	80
70Ex:30HP_80NMP	14	6	80

**Table 2:** Viscosity of used reagents at 25 °C.

Reagent	Viscosity, mPas
Ex8	151,000
HEMA-P	578
HEMA	5.74
NMP	1.81

**Table 3:** Parameters of the mathematical model obtained from optimization.

Parameter	Mathematical Model of Puncture Resistance RMS = 4.0, R^2^ = 0.95		Mathematical Model of Conductivity RMS = 3.8, R^2^ = 0.95	
	Value	*p*-Value ^1^	Value	*p*-Value ^1^
*b*_1_	−41.8	<0.001	92.8	<0.001
*b*_2_	−346	0.022	−93.2	0.001
*b*_3_	230	<0.001	−1282	0.008
*b*_12_	600	<0.001	-	-
*b*_13_	146	0.002	2228	0.034
*b*_23_	-	-	1480	0.022
*b*_123_	−1128	<0.001	-	-
*d*_12_	-	-	−41.5	0.025
*d*_13_	-	-	−1874	0.001
*d*_23_	2584	0.033	-	-

^1^ Parameters with *p*-value below α < 0.05 are statistically significant.

**Table 4:** Optimal photopolymerization mixture composition and conductivity, puncture resistance value obtained from the mathematical model and experiment.

Optimal Mixture Composition	Conductivity and Puncture Resistance	
	Mathematical Model	Experimental Value
X_NMP_ = 0.66 X_HEMA-P_ = 0.17 X_Ex8_ = 0.17	σ = 15.5 ± 1.3 mS·cm^−1^	σ = 15.7 ± 1.1 mS·cm^−1^
	F = 14.9 ± 0.9 N	F = 16.5 ± 1.4 N

## Data Availability

The data presented in this study are available upon request from the corresponding author.

## Supplementary Materials

The following are available online at https://www.mdpi.com/article/10.3390/polym13203495/s1, Figure S1: (a) Electrolyte, (b) water mass fraction in HPE, Figure S2: Residuals analysis (a) predicted vs. actual value for modeling of conductivity, (b) predicted vs. actual value for modeling of puncture resistance, Table 1: Parameters of the fitted EEC model.
