# Supplementary material for: Optimization of the Properties of Photocured Hydrogels for Use in Electrochemical Capacitors
Source: Polymers (Basel). 2021 Oct 12;13(20):3495. doi: 10.3390/polym13203495 (PMC8539391; doi:10.3390/polym13203495)
Supplement: Supplementary file 1 [file polymers-13-03495-s001.zip › ESI_after corrections.pdf]

Optimization of the properties of photocured hydrogels for use in electrochemical capacitors

Piotr Gajewski[1*], Aneta Lewandowska[1], Katarzyna Szcześniak[1], Grzegorz Przesławski[1] and Agnieszka Marcinkowska[1*]

[1] Institute of Chemical Technology and Engineering, Poznan University of Technology, Berdychowo 4, Poznan, 60-965, Poland; katarzyna.szczesniak@put.poznan.pl (K.S.), aneta.b.lewandowska@doctorate.put.poznan.pl (A.L.), grzegorz.przeslawski@doctorate.put.poznan.pl (G.P.)

* Correspondence: piotr.gajewski@put.poznan.pl (P.G.), agnieszka.marcinkowska@put.poznan.pl (A.M.)

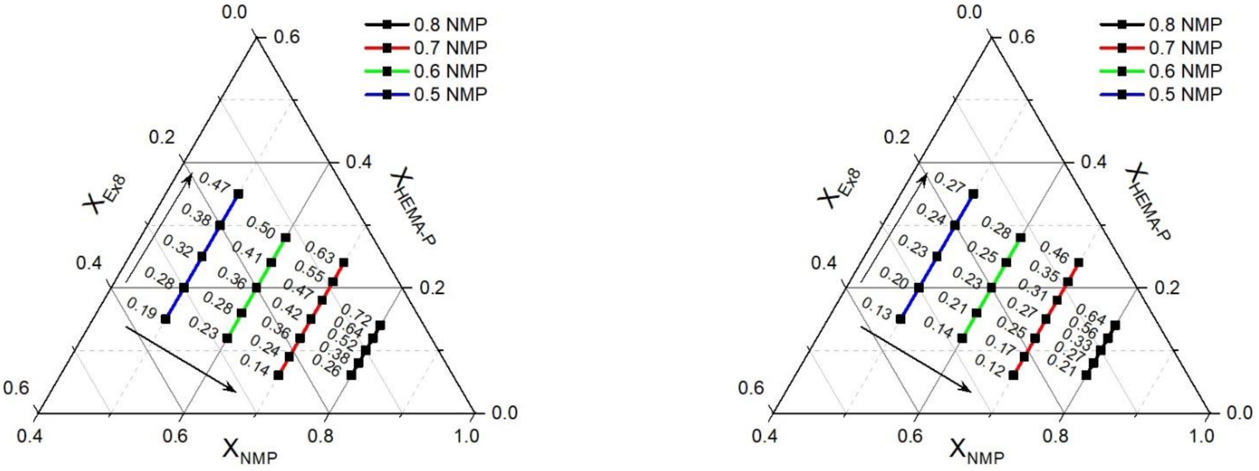

a) b)

Figure S1. (a) Electrolyte, (b) water mass fraction in HPE.

As can be seen (Figure S1a and S1b), the initial composition of the photocurable mixture has a significant effect on the sorption of water and electrolytes. For the same mixtures compositions, a lower water absorption could be observed. This may be due to the physical cross-linking between the water and the HEMA-P functional groups due to the formation of hydrogen bonds. During electrolyte sorption, NaOH is added (to maintain the pH = 6.9-7.0), which neutralizes HEMA-P and the hydrogen bonds disappear. As a result, the structure is no longer as densely cross-linked and higher electrolyte absorption is observed.

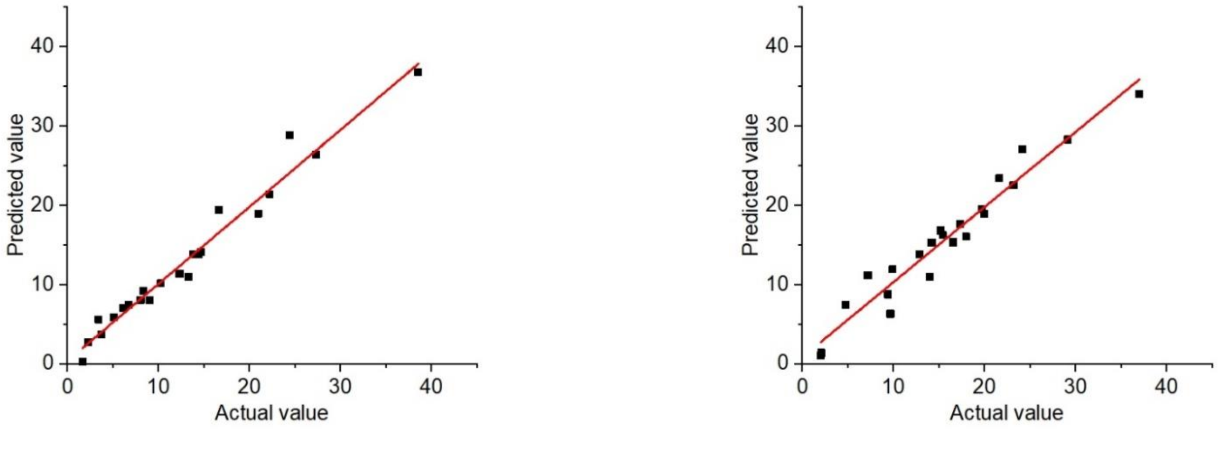

a) b)

Figure S2. Residuals analysis (a) predicted vs actual value for modelling of conductivity, (b) predicted vs actual value for modelling of puncture resistance.

As it could be seen (Figure S2a and S2b), a very good approximation of experimental data by the mathematical model was obtained. It is also confirmed by a high determination coefficient for both analysis, which is equal to R2=0.95.

Table S1. Parameters of fitted EEC model

		**HPE100**	**HPE300**
**R1, Ω**		0.518	0.802
**C1, F**		0.00144	0.00168
**R2, Ω**		0.0281	0.031
**Warburg area (diffusion resistance)**	**Rd, Ω**	0.503	0.527
	**t, s**	1.94	2.22
	**a, -**	0.97	0.99
